# A comparison of ^64^Cu-labeled bi-terminally PEGylated A20FMDV2 peptides targeting integrin α_ν_β_6_

**DOI:** 10.18632/oncotarget.28197

**Published:** 2022-02-16

**Authors:** Truc T. Huynh, Sreeja Sreekumar, Cedric Mpoy, Buck E. Rogers

**Affiliations:** ^1^Department of Radiation Oncology, Washington University School of Medicine, St. Louis, MO, USA; ^2^Department of Chemistry, Washington University, St. Louis, MO, USA

**Keywords:** integrin α_ν_β_6_, PEGylation, Copper-64, DOTA, PCTA

## Abstract

Expression of epithelial-specific integrin α_ν_β_6_ is up-regulated in various aggressive cancers and serves as a prognostic marker. Integrin-targeted PET imaging probes have been successfully developed and tested in the clinic. Radiotracers based on the peptide A20FMDV2 derived from foot-and-mouth disease virus represent specific and selective PET ligands for imaging α_ν_β_6_-positive cancers. The present study aims to describe the radiolabeling, *in vitro* and *in vivo* evaluation of a bi-terminally PEGylated A20FMDV2 conjugated with DOTA or PCTA for ^64^Cu radiolabeling. Stability studies showed radiolabeled complexes remained stable up to 24 h in PBS and human serum. *In vitro* cell assays in CaSki cervical cancer cells and BxPC-3 pancreatic cancer cells confirmed that the peptides displayed high affinity for α_v_β_6_ with K_d_ values of ~50 nM. Biodistribution studies revealed that [^64^Cu] Cu-PCTA-(PEG28)_2_-A20FMDV2 exhibited higher tumor uptake (1.63 ± 0.53 %ID/g in CaSki and 3.86 ± 0.58 %ID/g in BxPC-3 at 1 h) when compared to [^64^Cu]Cu-DOTA-(PEG28)_2_-A20FMDV2 (0.95 ± 0.29 %ID/g in CaSki and 2.12 ± 0.83 %ID/g in BxPC-3 at 1 h) . However, higher tumor uptake was accompanied by increased radioactive uptake in normal organs. Therefore, both peptides are appropriate for imaging α_ν_β_6_-positive lesions although further optimization is needed to improve tumor-to-normal-tissue ratios.

## INTRODUCTION

Integrins are a class of receptors that play essential roles in mediating cell adhesion, making transmembrane connections to the cytoskeleton, and modulating many intracellular signaling pathways [1, 2]. Because of their diverse functions, integrins have been studied extensively for decades, leading to the design and development of integrin antagonists in the treatment of multiple types of cancers. In recent years, α_ν_β_6_ has received much attention where its overexpression has been discovered to promote malignant behavior and tumor progression, resulting in poor prognosis and a striking reduction in survival rates for cancer patients [3–7]. α_ν_β_6_ has many regulatory functions in oncogenesis and its interaction with fibronectin and/or activation of TGFβ1 are known to lead to invasion and cancer migration [8, 9]. Increased expression levels of α_ν_β_6_ have been shown in various cancers including pancreatic, cervical, non–small cell lung cancer and oral squamous cell carcinoma [10–13].

Several α_ν_β_6_-targeting ligands have been identified and used in preclinical therapeutic and imaging studies. Hausner et al. used a 20-mer peptide (NAVPNLRGDLQVLAQKVART; A20FMDV2) that showed preferential binding to α_ν_β_6_ [14]. Another 20-mer peptide (RGDLATLRQLAQEDGVVGVR; H2009) isolated from a phage-display peptide library by panning on a lung adenocarcinoma cell line also binds to the restrictively expressed integrin α_ν_β_6_ [13]. Kimura et al. demonstrated that highly stable cystine knot peptides have potent and specific integrin α_ν_β_6_ binding for cancer detection [15]. The shared sequence of “RGDLXXL” (X represents unspecified amino acid) in the central binding region is key to high binding affinity and good selectivity towards α_ν_β_6_ integrin [16, 17]. Phage display libraries confirmed that RGD sequence followed by an LXXL motif is crucial in targeting α_ν_β_6_, while having minimal interactions with α_ν_β_3_, α_ν_β_5_, and α_IIb_β_3_ [16].

This study focuses on the 20-amino-acid, A20FMDV2, derived from the foot-and-mouth disease virus, which was used as a first-generation radiotracer for targeting α_ν_β_6_ *in vivo* [14]. A20FMDV2 was initially labeled with ^18^F and showed specific binding to α_ν_β_6_ both in *in vitro* cell assays and *in vivo* tumor-bearing models [18–20]. Though the initial lead compound A20FMDV2 exhibited good affinity towards integrin α_ν_β_6_, *in vivo* studies in tumor mouse models showed rapid excretion, metabolic breakdown, and unexpected high and persistent levels of radioactivity in non-target organs. To improve the pharmacokinetics of peptides, modifications such as cysteine amino acid substitutions, cyclization, PEGylation and incorporation of non-proteinogenic amino acid substitutes have been introduced [21–23]. Bi-terminal PEGylation of the peptide resulted in favorable *in vitro* and *in vivo* behavior concerning α_ν_β_6_ cell binding affinity and tumor uptake in mouse models [23]. This led to the first-in-human microdose study to assess the safety and pharmacokinetics of [^18^F]FBA-(PEG28)_2_-A20FMDV2 which confirmed the favorable performance of bi-terminally PEGylated peptide for identification of small lesions in primary sites as well as common sites of metastatic diseases [24].

However, radiolabeling of A20FMDV2 with ^64^Cu (T_1/2_ 12.7 h, β^+^ = 17%, β^−^ = 39%, EC = 43%, E_max_ = 0.656 MeV) would allow for easier radiochemical synthesis compared to ^18^F and allow for later imaging time points where contrast might be improved. Previous studies have used mono-PEGylated A20FMDV2 conjugated with various chelators and evaluated after radiolabeling with ^64^Cu [25, 26]. The goal of the present study is to use the optimized bi-terminally PEGylated A20FMDV2 conjugated with either DOTA (S-2-(4-isothiocyanatobenzyl)-1,4,7,10-tetraazacyclododecane-tetraacetic acid) or PCTA (3,6,9,15-tetraazabicyclo[9.3.1] pentadeca-1(15),11,13-triene-4-S-(4-isothiocyanatobenzyl)-3,6,9-triacetic acid) and evaluate these constructs *in vitro* and *in vivo* after radiolabeling with ^64^Cu. We chose to evaluate these two chelators because it is known that DOTA does not form the most kinetically stable complexes with copper and therefore we hypothesized that the PCTA peptide would have a better *in vivo* profile than the DOTA peptide.

## RESULTS

### Macrocyclic chelator integrin α_ν_β_6_ targetting peptides

**Figure 1:**
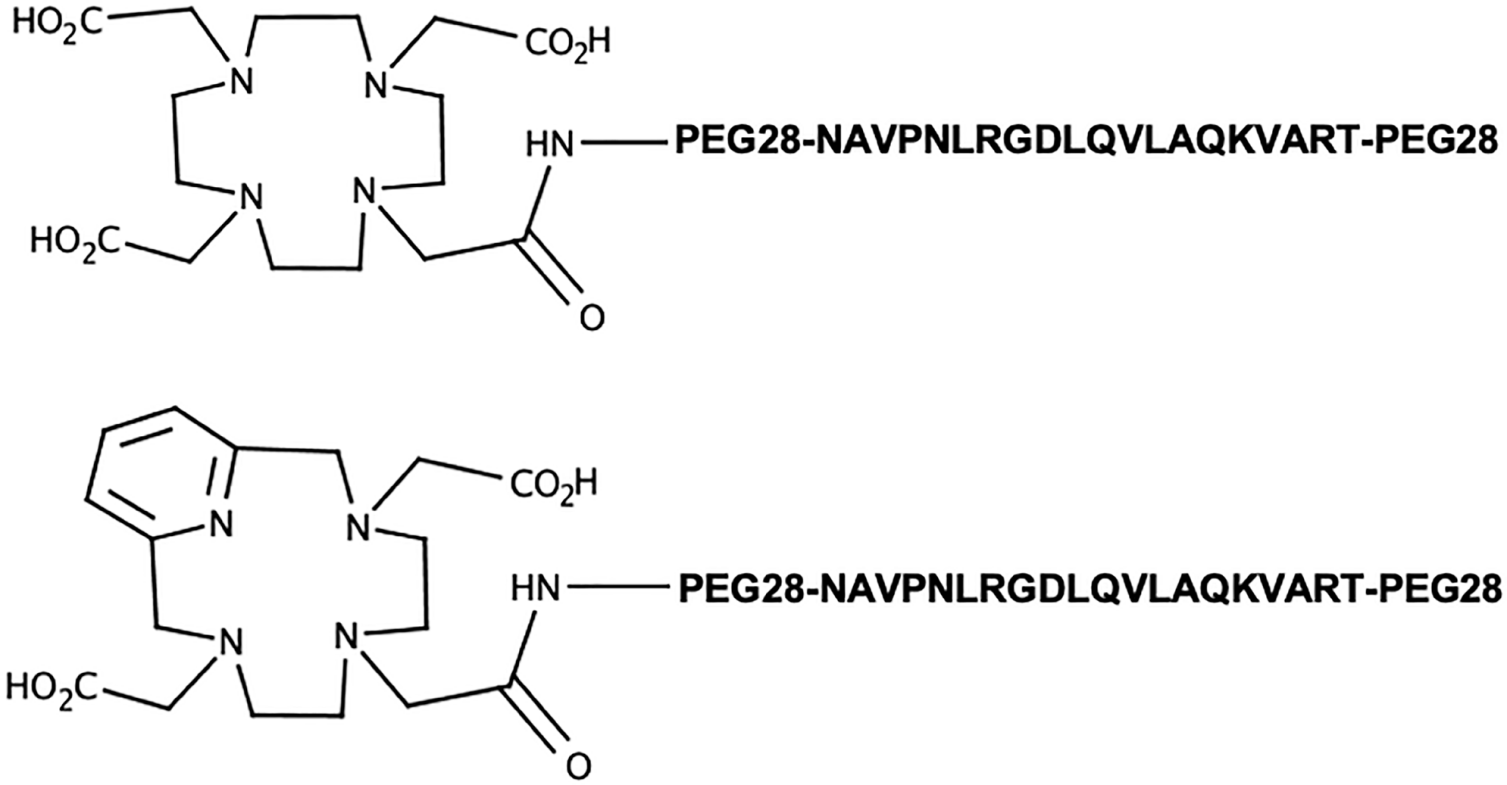
Structures of the α_ν_β_6_-targeting ligands investigated herein.

Bi-terminally PEGylated A20FMDV2 were custom synthesized and conjugated with DOTA and PCTA (Figure 1) and found to be >93 % pure by HPLC and confirmed by mass spectrometry.

### Radiochemistry

The radiolabeling results of the peptides with ^64^Cu is shown in Table 1. Radiolabeled products were obtained in high radiochemical purity (>95%) as determined by radio-HPLC and used without further purification. The molar activity of both radiolabeled peptides was 18.5 GBq/μmol. HPLC retention times were 8:05 and 8:37 min for [^64^Cu]Cu-DOTA-(PEG28)_2_-A20FMDV2 and [^64^Cu]Cu-PCTA-(PEG28)_2_-A20FMDV2, respectively (Supplementary Figure 1). Similarly, radio-TLCs demonstrated more than 95% radiochemical purity (Supplementary Figure 2).

**Table 1:** Summary of *in vitro* and *in vivo* studies for investigated α_ν_β_6_-targeting ligands

	^64^Cu-DOTA conjugate	^64^Cu-PCTA conjugate
**Labeling Conditions**	45°C for 1 h	80°C for 15 mins
**Molar Activity (GBq/μmol)**	18.5	18.5
**Labeling Efficiency**	>95%	>98%
**Human Serum Stability**	98.9% at 24 h	100% at 24 h
**K_d_ (nM)**	62.4 ± 3.7 (CaSki) 37.6 ± 5.4 (BxPC-3)	60.4 ± 6.7 (CaSki) 38.1 ± 4.7 (BxPC-3)
**B_max_ (# receptors per cell)**	0.75 ± 0.03 × 10^5^ (CaSki) 1.15 ± 0.10 × 10^5^ (BxPC-3)	0.73 ± 0.02 × 10^5^ (CaSki) 1.27 ± 0.02 × 10^5^ (BxPC-3)
**Tumor Uptake at 1 h p.i (%ID/g)**	0.95 ± 0.29 (CaSki) 2.12 ± 0.83 (BxPC-3)	1.63 ± 0.53 (CaSki) 3.86 ± 0.58 (BxPC-3)

### Serum stability

*In vitro* stability studies of all radiotracers were assessed by incubating in PBS 1X and human serum. There was less than 4% loss of ^64^Cu in PBS or human serum observed at 24 h (Supplementary Figure 3).

### Flow cytometry

Flow cytometry (Supplementary Figure 4) confirmed the expression level of integrin α_ν_β_6_ on CaSki and BxPC-3 cells with MFUs of 12.4 ± 0.1 and 11.1 ± 0.3 respectively when incubated the α_ν_β_6_-positive antibody as compared to the control antibody (0.4 ± 0.1 for CaSki cells and 1.2 ± 0.1 for BxPC-3 cells). Expression values on the SiHa and ME-180 cell lines were 0.4 ± 0.1 and 1.5 ± 0.1, respectively, demonstrating the relatively high expression level on CaSki and BxPC-3 cells.

### Cell binding and internalization

**Figure 2:**
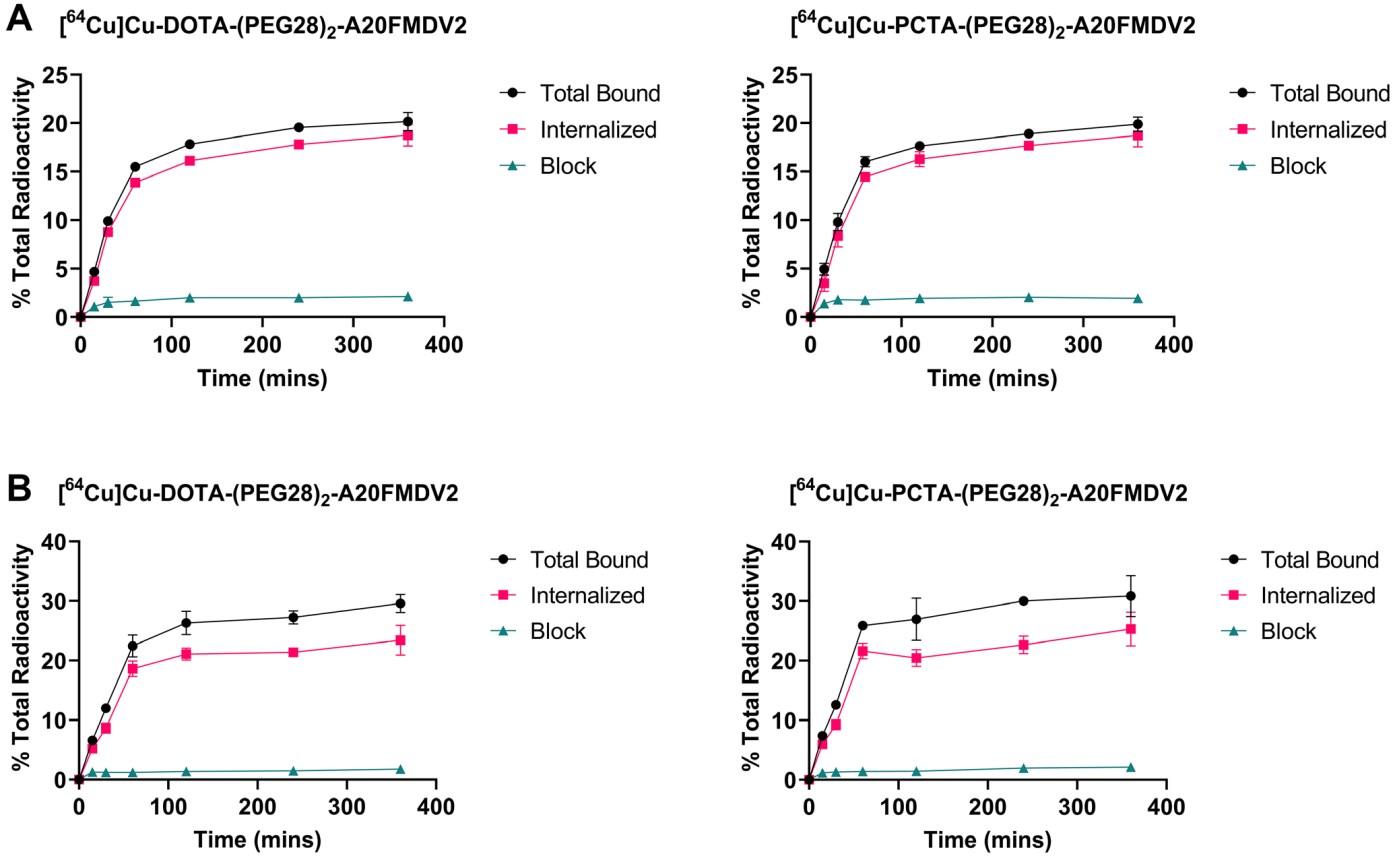
Representative binding and internalization curves of radiotracers *in vitro* in (**A**) CaSki cell line (**B**) BxPC-3 cell line. Total bound and internalization levels were shown as percent relative to total radioactivity. Data are presented as triplicates of mean ± SD.

Both radiolabeled peptides showed binding to α_ν_β_6_–expressing cell lines that were significantly inhibited by blocking with A20FMDV2, demonstrating α_ν_β_6_-specific binding (Figure 2). The binding was similar between the two peptides [^64^Cu]Cu-PCTA-(PEG28)_2_-A20FMDV2 and [^64^Cu]Cu-DOTA-(PEG28)_2_-A20FMDV2. The binding of both peptides in CaSki and BxPC-3 cells increased rapidly over a 2-h time frame and then plateaued for subsequent time points. More than 75% of the total bound radioactivity was internalized into the cells. Radiolabeled [^64^Cu]Cu-DOTA-(PEG28)_2_-A20FMDV2 peptide was internalized into the cells at a steady rate from 4% at 15 mins to 18% at 2 h in CaSki cells and from 5% at 15 mins to 20% at 2 h in BxPC-3 cells. Similar behavior was observed for [^64^Cu]Cu-PCTA-(PEG28)_2_-A20FMDV2 in which internalization increased from 4% at 15 mins to 18% at 2 h in CaSki cells, and from 6% at 15 mins to 23% at 2 h in BxPC-3 cells.

### Radioligand saturation binding

**Figure 3:**
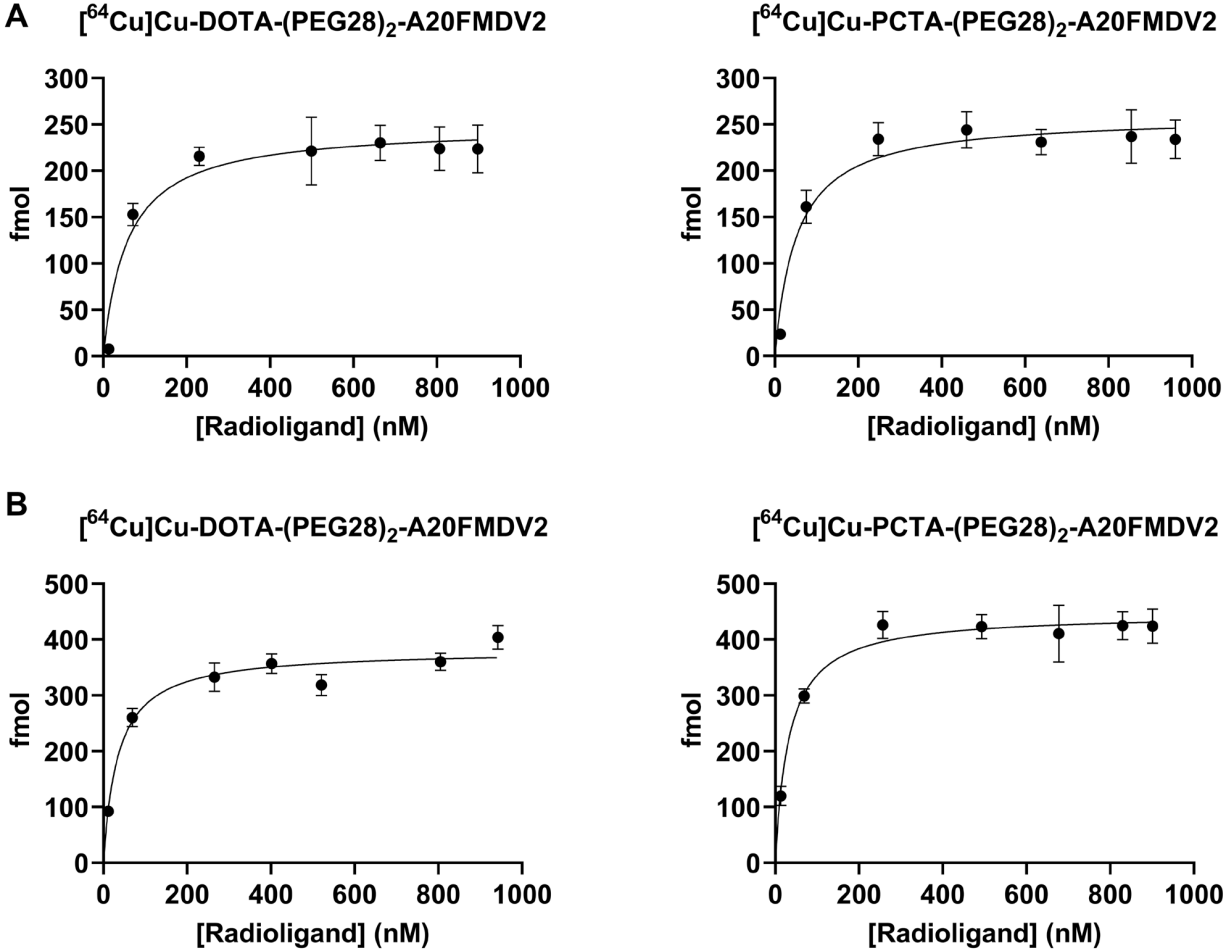
Representative saturation binding curves of radiotracers in (**A**) CaSki cell line (**B**) BxPC-3 cell line. Y-axis represents fmol of [^64^Cu]Cu-labeled bi-terminally PEGylated peptides bound to integrin α_ν_β_6_ -positive cells. Data are presented as triplicates of mean ± SD.

Receptor-binding affinity of the radiolabeled peptides to α_ν_β_6_ was evaluated on CaSki and BxPC-3 cell lines using a saturation binding assay at 4^°^C. Binding affinities were investigated by determining the equilibrium dissociation constant (K_d_) and the maximum binding capacity (B_max_) of radiolabeled conjugates to integrin α_ν_β_6_ -positive cells. Representative saturation binding curves in CaSki and BxPC-3 cell lines are shown in Figure 3. K_d_ values for [^64^Cu]Cu-DOTA-(PEG28)_2_-A20FMDV2 (62.4 ± 3.7 nM) and [^64^Cu]Cu-PCTA-(PEG28)_2_-A20FMDV2 (60.4 ± 6.7 nM) were similar for CaSki cells and slightly better when evaluated on BxPC-3 cells (37.6 ± 5.4 nM for [^64^Cu]Cu-DOTA-(PEG28)_2_-A20FMDV2 and 38.1 ± 4.7 nM for [^64^Cu]Cu-PCTA-(PEG28)_2_-A20FMDV2). The B_max_ was calculated to be about 0.75 × 10^5^ receptors per cell in CaSki cell line, whereas BxPC-3 has a maximum binding capacity of approximately 1.20 × 10^5^ receptors per cell for both radiotracers.

### Biodistribution studies

**Figure 4:**
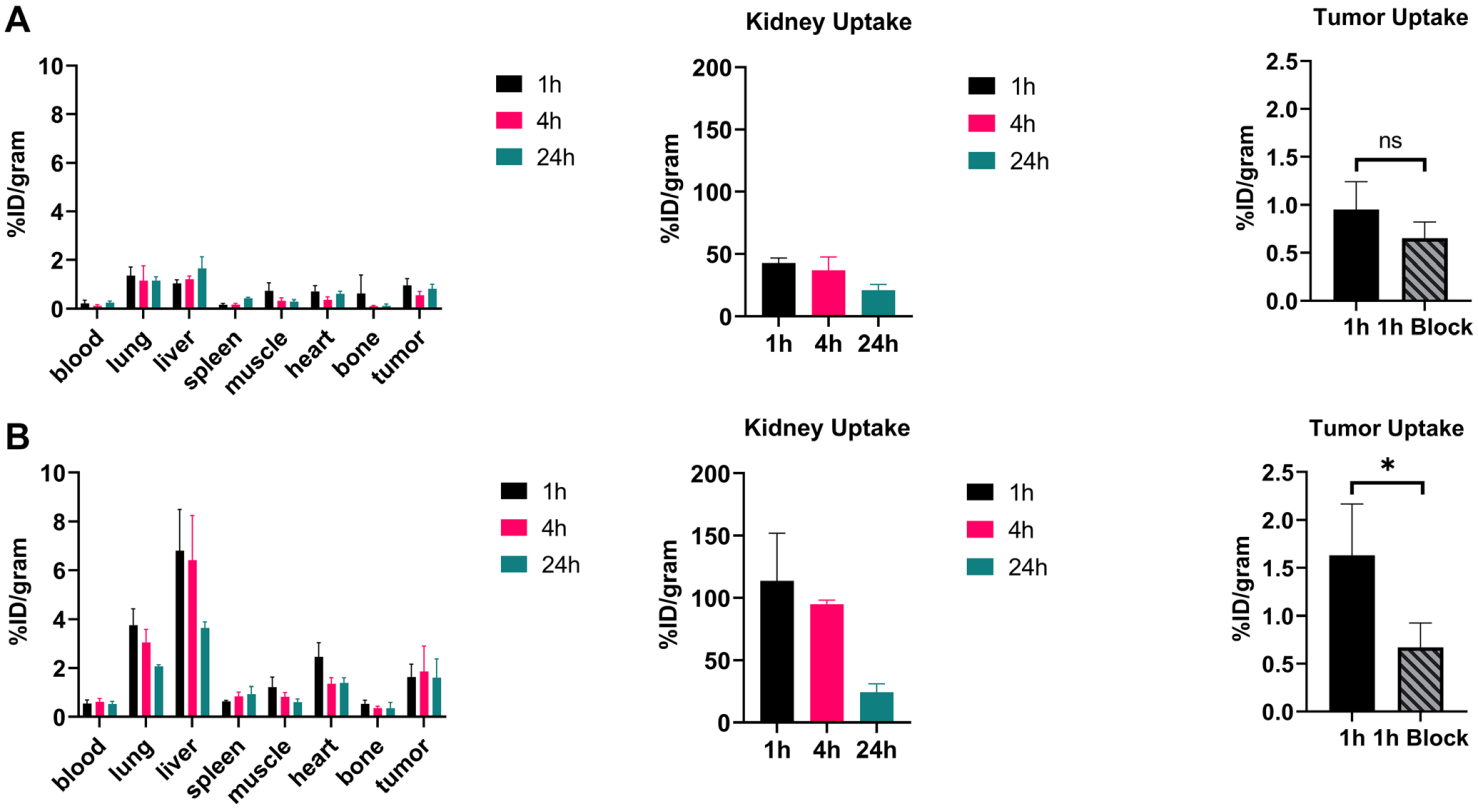
Biodistribution of ^64^Cu radiotracers in mice bearing α_ν_β_6_-expressing CaSki xenograft tumors (**A**) Radiotracer uptake of [^64^Cu]Cu-DOTA-(PEG28)2-A20FMDV2 (**B**) Radiotracer uptake of [^64^Cu]Cu-PCTA-(PEG28)2-A20FMDV2 in tumors and selected organs (%ID/g; bars = SD; tumors: *n* = 4 per time point) (ns: no significant, ^*^ *p* < 0.05, *T*-test).

Biodistribution of the radiolabeled peptides was studied at 1, 4, and 24 h post-injection. Figure 4 shows the biodistribution of both peptides in mice bearing CaSki xenografts. Both showed rapid blood clearance with only 0.26 ± 0.07 %ID/g for [^64^Cu]Cu-DOTA-(PEG28)_2_-A20FMDV2 and 0.53 ± 0.10 %ID/g for [^64^Cu]Cu-PCTA-(PEG28)_2_-A20FMDV2 remaining in the blood at 24 h. Overall accumulation of radioactivity in tumors and non-target organs was higher for [^64^Cu]Cu-PCTA-(PEG28)_2_-A20FMDV2 than [^64^Cu]Cu-DOTA-(PEG28)_2_-A20FMDV2. High kidney uptake was observed for [^64^Cu]Cu-PCTA-(PEG28)_2_-A20FMDV2 at 113.58 ± 38.31 %ID/g at 1 h p.i, which decreased to 24.38 ± 6.54 %ID/g at 24 h. On the other hand, high and persistent retention of radioactive uptake in kidney was observed for [^64^Cu]Cu-DOTA-(PEG28)_2_-A20FMDV2, as evidenced by an accumulation of 42.72 ± 4.03 %ID/g at 1 h p.i followed by a slight decrease to 37.04 ± 10.60 %ID/g at 4 h and remained at 20.99 ± 4.67 %ID/g at 24 h. Despite [^64^Cu]Cu-PCTA-(PEG28)_2_-A20FMDV2 having higher kidney uptake at the initial time points, both the peptides eventually decreased to similar levels at 24 h.

**Figure 5:**
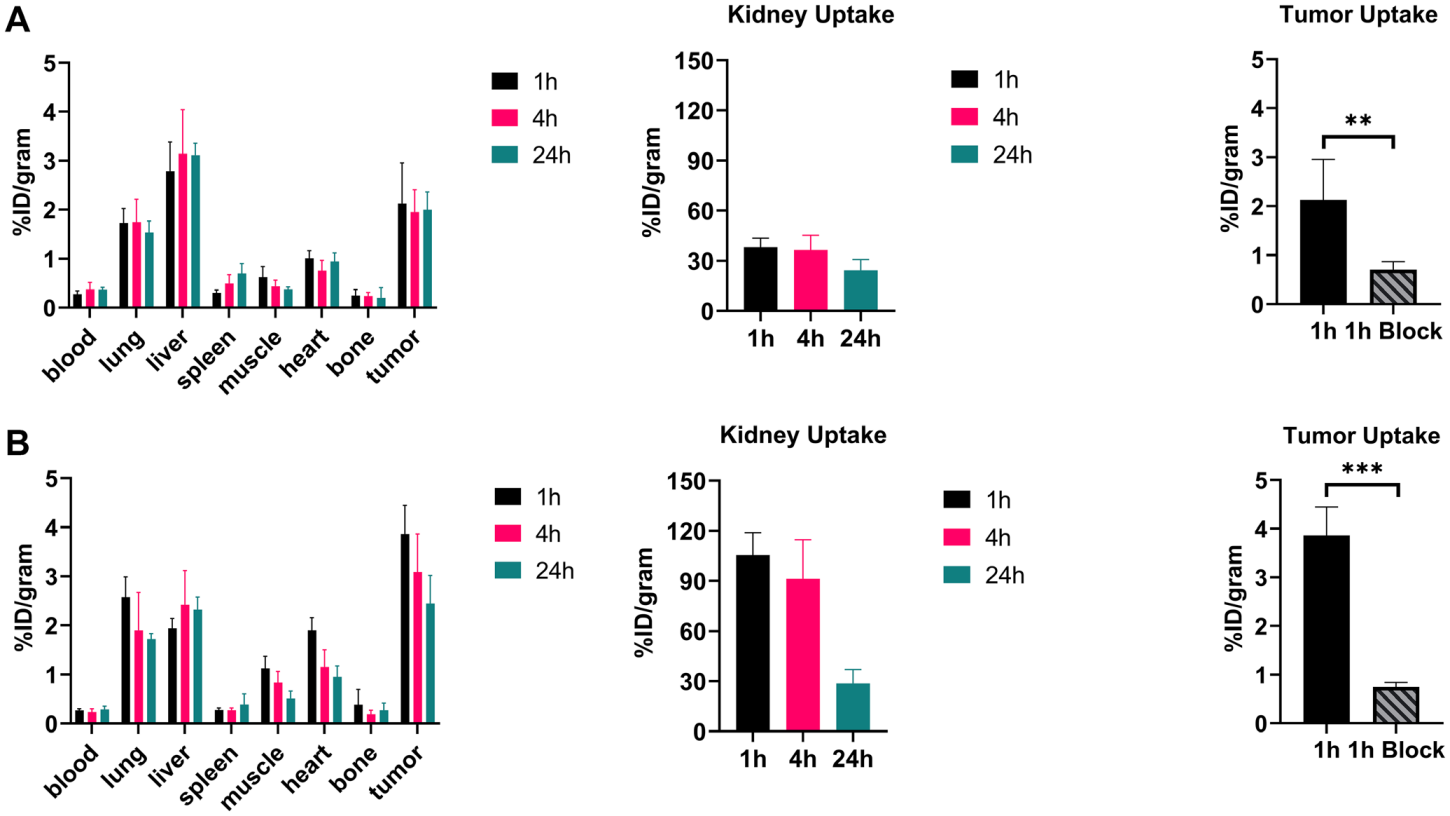
Biodistribution of ^64^Cu radiotracers in mice bearing α_ν_β_6_-expressing BxPC-3 xenograft tumors (**A**) Radiotracer uptake of [^64^Cu]Cu-DOTA-(PEG28)2-A20FMDV2 (**B**) Radiotracer uptake of [^64^Cu]Cu-PCTA-(PEG28)2-A20FMDV2 in tumors and selected organs (%ID/g; bars = SD; tumors: *n* = 4 per time point) (^**^ *p* < 0.01, ^***^ *p* < 0.001, *T*-test).

Biodistribution profiles in BxPC-3 tumor-bearing mice were similar to those of CaSki (Figure 5). Tumor uptake of [^64^Cu]Cu-PCTA-(PEG28)_2_-A20FMDV2 (3.86 ± 0.58 %ID/g) was higher than that of [^64^Cu]Cu-DOTA-(PEG28)_2_-A20FMDV2 (2.12 ± 0.83 %ID/g) at 1 h p.i and exhibited good tumor retention with a slight decrease at the 4 h time point (3.09 ± 0.77 %ID/g) and an uptake of 2.45 ± 0.57 %ID/g at 24 h. Despite showing higher accumulation in normal organs compared to [^64^Cu]Cu-DOTA-(PEG28)_2_-A20FMDV2, [^64^Cu]Cu-PCTA-(PEG28)_2_-A20FMDV2 shows shorter retention of activity in normal organs, as indicated by kidney uptake decreasing from 105.39 ± 13.58 %ID/g at 1 h to 28.65 ± 8.45 %ID/g at 24 h.

Between the two radiolabeled peptides, tumor-to-normal-tissue ratios were generally similar for the two peptides (Supplementary Tables 1 and 2). Despite the fact that [^64^Cu]Cu-PCTA-(PEG28)_2_-A20FMDV2 has a two-fold higher tumor uptake *in vivo*, it was compensated by increased radioactive uptake in non-target organs. Blocking studies with unlabeled A20FMDV2 confirmed the specific uptake of the peptides in integrin α_ν_β_6_-positive tumors. Injection of excess unlabeled peptide resulted in a statistically significant reduction in binding of [^64^Cu]Cu-PCTA-(PEG28)_2_-A20FMDV2 in CaSki tumors at 1 h p.i (0.67 ± 0.26 %ID/g with blocking compared to 1.63 ± 0.53 %ID/g without blocking, *p* = 0.02). There was a reduction in binding of [^64^Cu]Cu-DOTA-(PEG28)_2_-A20FMDV2, but this did not reach significance (0.65 ± 0.17 %ID/g with blocking compared to 0.95 ± 0.29 %ID/g without blocking, *p* = 0.12). In the BxPC-3 tumor mouse model, blocking resulted in significantly reduced uptake of both radiotracers with 0.71 ± 0.16 %ID/g in tumors compared to 2.12 ± 0.83 %ID/g for [^64^Cu]Cu-DOTA-(PEG28)_2_-A20FMDV2 (*p* = 0.005) and 0.75 ± 0.09 %ID/g compared to 3.86 ± 0.58 %ID/g for [^64^Cu]Cu-PCTA-(PEG28)_2_-A20FMDV2 (*p* < 0.001). Normal organs where significant (*p* < 0.05) blocking occurred include the lung, kidney, muscle, and heart and indicate expression of α_ν_β_6_ in these tissues (Supplementary Tables 3 and 4).

### PET/CT imaging

**Figure 6:**
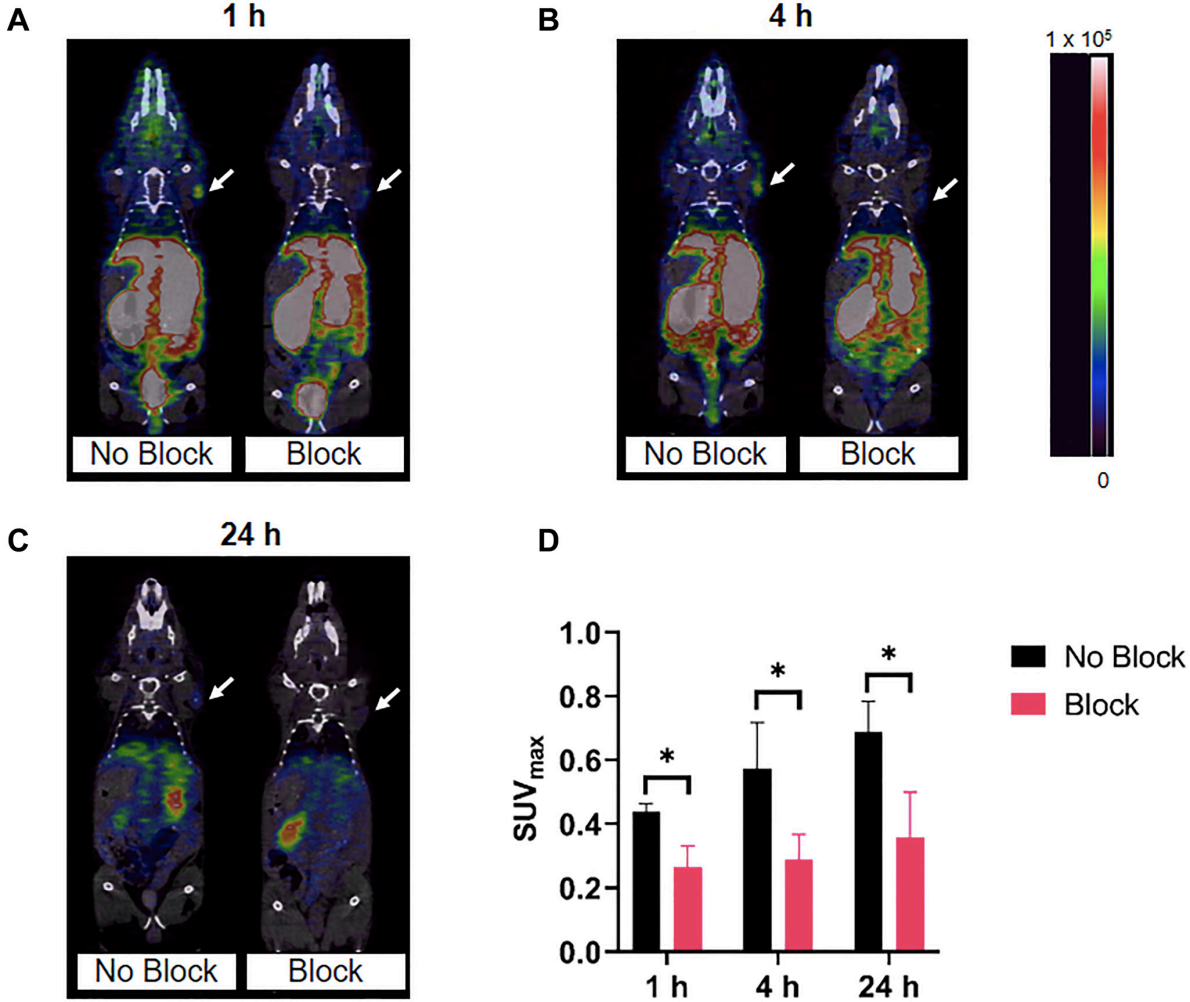
Representative micro-PET/CT co-registration images of [^64^Cu]Cu-DOTA-(PEG28)_2_-A20FMDV2 in athymic nude mice bearing BxPC-3 tumors (*n* = 3). PET imaging was performed at (**A**) 1 h, (**B**) 4 h, (**C**) 24 h after intravenous injection of 3.7 MBq of [^64^Cu]Cu-DOTA-(PEG28)_2_-A20FMDV2 with or without blocking. White arrows indicate the positions of the tumor xenografts. The scale bar unit is Bq/mL (**D**) Maximum standared uptake values of imaged tumors (^*^ *p* < 0.05, *T*-test).

**Figure 7:**
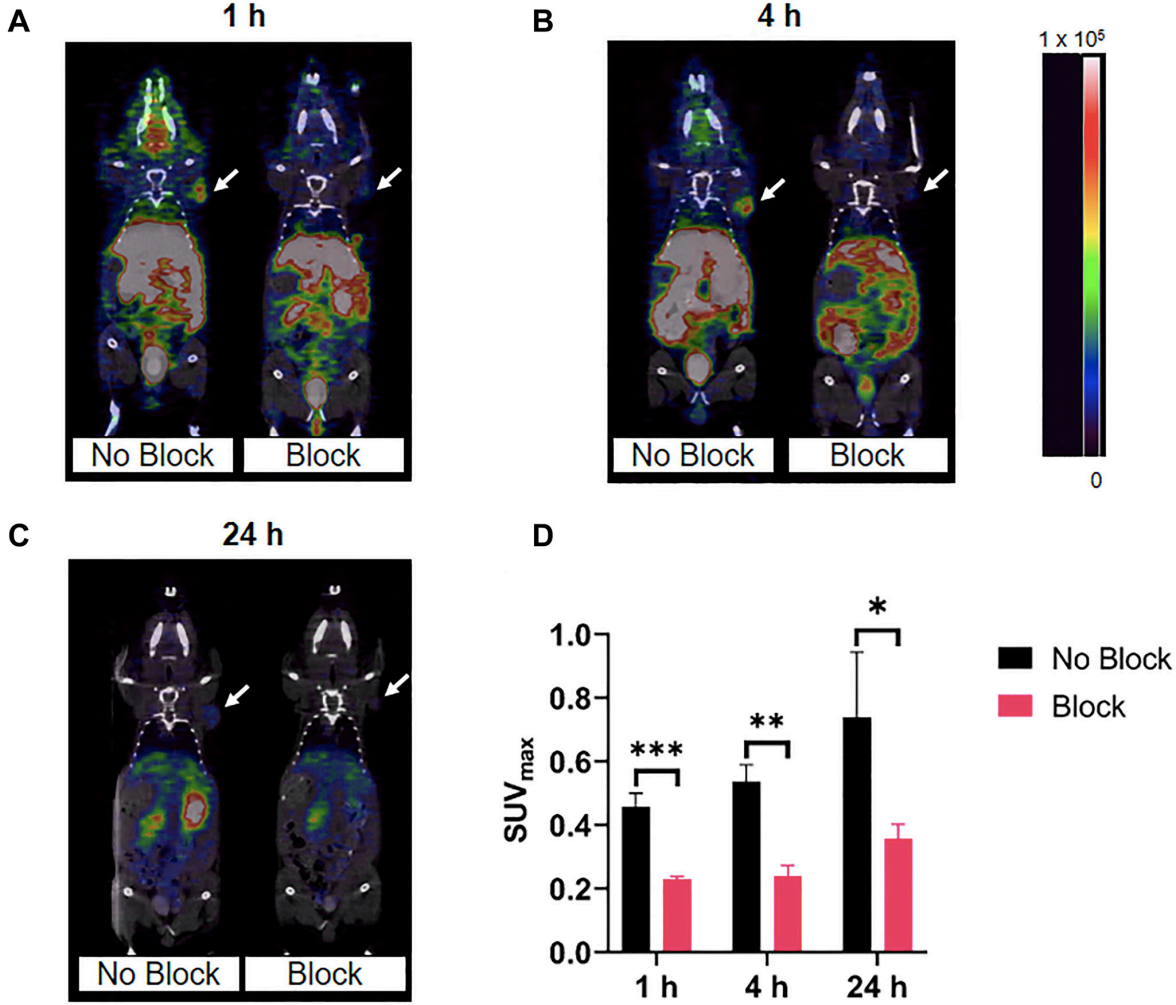
Representative micro-PET/CT co-registration images of [^64^Cu]Cu-PCTA-(PEG28)_2_-A20FMDV2 in athymic nude mice bearing BxPC-3 tumors (*n* = 3). PET imaging was performed at (**A**) 1 h, (**B**) 4 h, (**C**) 24 h after intravenous injection of 3.7 MBq of [^64^Cu]Cu-PCTA-(PEG28)_2_-A20FMDV2 with or without blocking. White arrows indicate the positions of the tumor xenografts. The scale bar unit is Bq/mL (**D**) Maximum standard uptake values of imaged tumors (^*^ *p* < 0.05, ^**^ *p* < 0.01, ^***^ *p* < 0.001, *T*-test).

Small animal PET/CT images of [^64^Cu]Cu-DOTA-(PEG28)_2_-A20FMDV2 and [^64^Cu]Cu-PCTA-(PEG28)_2_-A20FMDV2 at 1, 4, and 24 h are shown in Figures 6 and 7. These figures show a representative single slice of the coronal PET/CT images for mice bearing BxPC-3 xenografts. These images show good tumor uptake of both radiolabeled constructs at all three time points that was inhibited by an excess of blocking agent. Images show clearance of the radioactivity from the normal tissues and tumor over time. Image analysis of the tumors shows that SUV_max_s increase from 0.44 ± 0.02 at 1 h to 0.57 ± 0.15 at 4 h to 0.69 ± 0.09 at 24 h for [^64^Cu]Cu-DOTA-(PEG28)_2_-A20FMDV2 due to the more rapid clearance of radioactivity from normal tissue compared to tumors. A similar trend was observed for [^64^Cu]Cu-PCTA-(PEG28)_2_-A20FMDV2 which showed SUV_max_s increasing from 0.46 ± 0.04 at 1 h to 0.54 ± 0.05 at 4 h to 0.74 ± 0.21 at 24 h. In addition, tumor specificity is confirmed as the mice that received blocking agent had significantly smaller SUVs (*p* < 0.044) compared to the non-blocked mice.

## DISCUSSION

Integrin α_ν_β_6_, expressed exclusively in epithelial cells, lends itself to be an excellent molecular target for imaging and therapy as it is not expressed in normal adult epithelia but is expressed under special wound healing conditions and in cancer [3–7]. In fact, overexpression of integrin α_ν_β_6_ has been observed in various forms of cancer including those of pancreatic, cervical, non–small cell lung cancer and oral squamous cell carcinoma [10–13]. Previous research has shown the correlation between α_ν_β_6_ expression with more aggressive disease, reduced survival rates and increased chance of metastasis. Therefore, the design and development of α_ν_β_6_-targeting peptides that can serve as both imaging and therapeutic agents would be valuable. In this regard, the 20-mer peptide, A20FMDV2, has been extensively studied due to its specific binding toward integrin α_ν_β_6_ [14]. The lead peptide was optimized and radiolabeled with ^18^F for imaging with a bi-terminal PEG construct used for first-in-human studies [24]. The purpose of this study was to evaluate A20FMDV2 for radiolabeling with copper for potential imaging or therapy. Previous studies investigated ^64^Cu labeling, only with mono-PEGylation, which is not optimal [25, 26]. Therefore, this study employed the bi-terminally PEGylated peptide conjugated to DOTA or PCTA for radiolabeling with ^64^Cu. The two peptides are compared *in vitro* and *in vivo*.

All radiotracers were radiolabeled in good radiochemical purity (>95 %) at a molar activity of 18.5 GBq/μmol (0.50 Ci/μmol). The molar activity of the DOTA conjugated peptide is comparable to previously published results by Hu et al. (0.48 Ci/μmol) and Hausner et al. (0.58–0.60 Ci/μmol) despite the additional PEG28 [25, 26]. This indicates that the introduction of another PEG28 does not affect the complexation of ^64^Cu with DOTA. Labeling of the PCTA peptide at 45^°^C resulted in 72% labeling; therefore we increased the temperature to 80^°^C for 15 mins to achieve >95% labeling. It is not clear why higher temperatures are needed as other studies have showed efficient ^64^Cu-labeling of PCTA conjugates at room temperature [27, 28], while some have used temperatures >60^°^C [29, 30]. While the *in vitro* serum stability cannot predict *in vivo* degradation from the liver and other organs, it can be used as a first screen and showed that both peptides were stable enough to warrant further *in vivo* investigation.

Similar to Hu et al., more than 75% of bound radioactivity of the two chelates were internalized by 1 h in both CaSki and BxPC-3 cells [25]. While the total amount of bound radioactivity at 1 h was less than 60% observed by Hu et al. for the [^64^Cu]Cu-DOTA labeled A20FMDV2 peptide, this could be due to the use of different cell lines or the additional PEG28 in our studies [25]. The saturation binding showed good affinity of 30–60 nM for both peptides when evaluated in CaSki or BxPC-3 cells. This is generally lower than the reported 1.73 ± 0.46 nM for [^111^In]In-DTPA-A20FMDV2, which can be due to the introduction of PEGylation onto the original peptide [31]. The B_max_ values for BxPC-3 were twice as higher for CaSki cells, which is supported by the higher tumor uptake of radiotracers in BxPC-3 compared to CaSki tumor models.

It was observed that [^64^Cu]Cu-PCTA-(PEG28)_2_-A20FMDV2 had higher tumor uptake than [^64^Cu]Cu-DOTA-(PEG28)_2_-A20FMDV2 in both CaSki and BxPC-3 tumor-bearing mice. The tumor uptake of [^64^Cu]Cu-PCTA-(PEG28)_2_-A20FMDV2 (3.86 ± 0.58 %ID/g) in BxPC-3 tumor mouse model is slightly lower than the uptake of 4.7 ± 0.9 %ID/g achieved by [^18^F]FBA-(PEG28)_2_-A20FMDV2 [23] and is higher that of [^64^Cu]Cu-DOTA-(PEG28)_2_-A20FMDV2 (2.12 ± 0.83 %ID/g), which agrees with the B_max_ data. Tumor uptake was specific for all of the constructs except for [^64^Cu]Cu-DOTA-(PEG28)_2_-A20FMDV2 in the CaSki tumors which is likely due to the lower expression of α_ν_β_6_ in this model. PET imaging in mice bearing BxPC-3 tumors confirmed the specific tumor targeting of both radiotracers towards integrin α_ν_β_6_ which indicates their potential as imaging agents for integrin α_ν_β_6_. However, the higher tumor uptake of [^64^Cu]Cu-PCTA-(PEG28)_2_-A20FMDV2 compared to [^64^Cu]Cu-DOTA-(PEG28)_2_-A20FMDV2 that was observed in the biodistribution studies was not seen in the PET imaging studies as the SUVs were similar at all time points. This may be due to the greater amount of mass of peptide (1–2 μg for imaging vs. 0.1–0.2 μg for biodistribution) that was injected for the imaging studies which may have partially blocked tumor uptake.

Overall, uptake in normal organs of [^64^Cu]Cu-PCTA-(PEG28)_2_-A20FMDV2 was generally higher than that of [^64^Cu]Cu-DOTA-(PEG28)_2_-A20FMDV2 at the earlier time points (1 and 4 h), especially in the kidney. The increased kidney uptake of [^64^Cu]Cu-PCTA-(PEG28)_2_-A20FMDV2 may be due to its +2 charge compared to the +1 charge of [^64^Cu]Cu-DOTA-(PEG28)_2_-A20FMDV2. Cationic portions of peptides may form electrostatic interactions with negative surface charge of proximal tubular cells of kidney, resulting in the trapping of the radiotracers and their metabolites in the tubular cells [32, 33]. Higher uptake in other tissues for [^64^Cu]Cu-PCTA-(PEG28)_2_-A20FMDV2 and in particular the liver may be due to the higher lipophilicity of PCTA compared to DOTA. In addition, there may be expression of α_ν_β_6_ in normal organs such as the lung, kidney, muscle, and heart due to inhibition of radiolabeled peptide uptake by an excess of unlabeled peptide (Supplementary Tables 3 and 4). Previous pre-clinical studies have not investigated expression in normal organs, however, clinical evaluation of similar peptides has shown low uptake in the brain, bone, liver, and lung indicating the potential value of imaging metastases in these sites.

In conclusion, [^64^Cu]Cu-PCTA-(PEG28)_2_-A20FMDV2 and [^64^Cu]Cu-DOTA-(PEG28)_2_-A20FMDV2 bound specifically to α_ν_β_6_ on tumor cells both *in vitro* and *in vivo*. While *in vivo* tumor uptake [^64^Cu]Cu-PCTA-(PEG28)_2_-A20FMDV2 was superior to that of [^64^Cu]Cu-DOTA-(PEG28)_2_-A20FMDV2, the normal tissue uptake of [^64^Cu]Cu-PCTA-(PEG28)_2_-A20FMDV2 was also greater. Thus, both peptides have similar tumor to normal tissue ratios and would be equally good for imaging and therapy, which contradicts our hypothesis that [^64^Cu]Cu-PCTA-(PEG28)_2_-A20FMDV2 would be superior. Future work will focus on modifications of the peptides to improve affinity and tumor to normal tissue ratios by possibly cyclizing the peptide and substituting more hydrophilic amino acids in positions that do not adversely affect binding.

## MATERIALS AND METHODS

### General information

All solvents and reagents were purchased from Sigma-Aldrich (St. Louis, MO, USA) or Fisher Scientific (Pittsburgh, PA, USA) and used as received unless stated otherwise. All solutions and buffers were prepared using HPLC-grade water. Peptides were custom synthesized and characterized by AnaSpec (Fremont, CA, USA). Stock solutions of the peptides (1 nmol/μl) were prepared in HPLC-grade water and stored at −20^°^C before use. Non-PEGylated A20FMDV2 peptide served as blocking agent. Radio-TLCs employed Whatman 60 Å silica gel thin-layer chromatography (TLC) plates and were analyzed using a Bioscan 200 imaging scanner (Bioscan, Inc., Washington, DC, USA). Reversed-phase high-pressure liquid chromatography (HPLC) was used to evaluate the radiolabeling efficiency. HPLC utilized a two-solvent system: water (0.05% trifluoroacetic acid (TFA)) and acetonitrile (0.05% TFA). The system was equipped with UV absorbance detectors (UV, 220 and 280 nm), a NaI radiotracer detector and a photodiode array detector. HPLC analysis of peptides used Kinetex (Phenomenex) C-18 column (5 μm, 4.6 × 150 mm I.D.).

### Cell culture

CaSki and BxPC-3 cell lines purchased from ATCC (Manassas, VA, USA) were revived from liquid nitrogen storage and cultured at 37^°^C, 5% CO_2_. The media for CaSki cells contained DMEM, 10% heat-inactivated fetal bovine serum (FBS) (Gibco), and 10 mM HEPES. The media for BxPC-3 cells contained RPMI 1640, 10% FBS, and 10 mM HEPES. Cell-labeling solutions and biodistribution samples were analyzed on a Beckman Gamma 8000 counter containing a NaI crystal (Beckman Instruments, Inc., Irvine, CA, USA).

### Radiochemical synthesis of [^64^Cu]Cu-DOTA-(PEG28)_2_-A20FMDV2 and [^64^Cu]Cu-PCTA-(PEG28)_2_-A20FMDV2

^64^Cu was produced from ^64^Ni(p,n)^64^Cu nuclear reaction on enriched ^64^Ni on a TR-19 biomedical cyclotron (Advanced Cyclotron Systems, Inc. - Canada) at Mallinckrodt Institute of Radiology, Washington University School of Medicine, and purified with an automated system using standard procedures [34, 35]. The resulting activity was diluted in 0.1M HCl at a specific activity ranging from 300 to 2000 mCi/μg. An aliquot of 2 μl DOTA-(PEG28)_2_-A20FMDV2 peptide (2 nmol) was diluted to 100 μl with 0.1M NH_4_OAc (pH 5.5). A stock solution of ^64^Cu in 0.1M HCl was diluted ten-fold with 0.1M NH_4_OAc (pH 5.5) for radiolabeling and 1mCi of ^64^Cu was added to the reaction mixture. The reaction mixture was incubated at 45^°^C for 1 h. The product was evaluated for radiochemical purity by radio-HPLC and TLC with a mobile phase of 50 mM DTPA. The radiolabeled complex remained at the origin in the TLC system, while the free ^64^Cu moved with solvent front.

The synthesis of [^64^Cu]Cu-PCTA-(PEG28)_2_-A20FMDV2 was similar to [^64^Cu]Cu-DOTA-(PEG28)_2_-A20FMDV2 except that reaction solution was incubated at 80^°^C for 15 mins. Quality control was performed by TLC and HPLC as described above.

### Serum stability

100 μl of human serum albumin (HSA 1g/ml) or 1X Phosphate Buffered Saline (PBS) was prepared in Eppendorf tubes, followed by the addition of 100 μl of ^64^Cu-radiolabeled peptides. The resulting reaction mixtures were incubated at 37^°^C with moderate agitation. At time points of 1, 4 and 24 h, aliquots (0.5 μl) were withdrawn from the sample and spotted on TLC plates. Radio-TLCs with a mobile phase of 50 mM diethylenetriamine pentaacetate (DTPA) was performed to evaluate the fraction of intact radiotracer at specific time points.

### Flow cytometry

Wash buffer of PBS 1X, 0.1% BSA, and 0.1% sodium azide was prepared. Confluent cells were harvested and re-suspended in wash buffer. Aliquots of 3 × 10^5^ cells in 96-well-plate were incubated with 100 μl primary Ab 10D5 (α_ν_β_6_ positive) (10 μg/mL in FACS buffer) at room temperature for 1 h, 500 rpm. After washing the cells three times with wash buffer, cells were incubated in 100 μl of Alexa Fluor 488 goat-anti-mouse (10 μg/mL diluted to 1:50 in FACS buffer) for 30 mins at room temperature. Cells were then rinsed and re-suspended in a final volume of 200 μl wash buffer. In control samples, the primary Ab 10D5 antibody was replaced by IgG2a Isotype Control (α_ν_β_6_ negative). Flow cytometric analysis was performed using a FACScan flow cytometer and data presented as mean fluorescence units (MFUs).

### Cell binding and internalization

For ^64^Cu cell-binding experiments, a method similar to that described by Geissler et al. was used [36]. Briefly, 3.7 KBq (0.1μCi) aliquots of [^64^Cu]Cu-DOTA-(PEG28)_2_-A20FMDV2 or [^64^Cu]Cu-PCTA-(PEG28)_2_-A20FMDV2 was added to a cell suspension of either CaSki or BxPC-3 cells in PBS 1X (pH 7.4; 2.0 × 10^6^ cells in 50 μl). Assay tubes were pretreated with 5% bovine serum albumin (5% wt/v in PBS) to block non-specific binding. Reaction mixtures were incubated at various time points (15 mins, 30 mins, 1 h, 2 h, 4 h and 6 h) with moderate shaking to prevent cell settling. Cells were centrifuged and supernatant was aspirated from cell pellets. The pellets underwent additional washes with 0.5 mL ice-cold PBS (3 times) and the fraction of bound radioactivity was measured with a gamma counter. To determine the fraction of internalized radiotracers, cells were treated with 2 × 0.5 mL of 20 mM sodium acetate (pH 4.0) for 5 mins to remove surface-bound radioactivity. Following centrifugation, cells were washed with 0.5 mL ice-cold PBS and counted for activity. To determine the specific binding towards α_ν_β_6_, non-PEGylated peptide (20 μg) was added 10 mins before the addition of radiotracers as a blocking agent. The experiments were performed in triplicate.

### Radioligand saturation binding

A cell suspension of 1.0 × 10^8^, either CaSki or BxPC-3 cells in 5 mL PBS was prepared. The non-PEGylated peptide was prepared to 10 μg/100 μl in PBS 1X as a blocking agent. Assay tubes were pretreated with 5% bovine serum albumin (5% wt/v in PBS) to block non-specific binding. A solution of 100 μL cells (2 × 10^6^ cells) and 100 μl of blocking or PBS were added to each assay tube, followed by a series of ^64^Cu-radiolabeled peptide concentrations that ranged between 5 nM and 1000 nM. Tubes were slightly agitated and placed on ice for 1 h. After incubation, tubes were centrifuged for 3 mins, and the supernatant was removed. Cell pellets were washed with ice-cold PBS three times and measured for activity on a gamma counter to determine the amount of surface-bound radioactivity. The experiments were done in triplicate in the presence and absence of a 1000-fold excess blocking agent. Saturation binding curves were generated with x-axis as the molarity of radiolabeled peptides vs. y-axis as specific binding in fmol. Curves were fitted with GraphPad Prism 9 to find the equilibrium constant (K_d_) and the maximum binding capacity (B_max_).

### Biodistribution studies

Animals were supplied by Charles River Laboratories (Wilmington, MA, USA), and were handled in compliance with the Guidelines for Care and Use of Research Animals established by the Division of Comparative Medicine and the Animal Studies Committee of Washington University School of Medicine under protocol #20–0214. Female athymic nude mice were inoculated on the right flank with 10 × 10^6^ CaSki or 5 × 10^6^ BxPC-3 cells. Tumors were allowed to grow until they approached approximately 100 mm^3^ in volume. Radiolabeled peptides were diluted in saline to a dose of 0.37 MBq (10 μCi) per 100 μl. Animals were injected intravenously with 0.37 MBq (10 μCi) of [^64^Cu]Cu-DOTA-(PEG28)_2_-A20FMDV2 or [^64^Cu]Cu-PCTA-(PEG28)_2_-A20FMDV2 peptides and sacrificed by cervical dislocation at 1, 4, and 24 h. For blocking experiments, non-PEGylated peptide (100 μg in 100 μL saline) was injected ten minutes before radiotracer. Blood, lung, liver, spleen, kidney, muscle, heart, bone, and tumor were harvested. The amount of radioactivity in each organ was determined by gamma counting, and the percent injected dose per gram of tissue (% ID/g) was calculated.

### PET/CT imaging

BxPC-3 cells (5 × 10^6^) were inoculated in the right shoulder of female athymic nude mice 3–4 weeks before PET imaging sessions. All the experiments involving animals followed the Guidelines for Care and Use of Research Animals established by the Division of Comparative Medicine and the Animal Studies Committee of Washington University School of Medicine under protocol #20–0214. On the day of the experiment, mice were injected intravenously with 3.7 MBq (100 μCi) of [^64^Cu]Cu-DOTA-(PEG28)_2_-A20FMDV2 or [^64^Cu]Cu-PCTA-(PEG28)_2_-A20FMDV2 peptides. Mice were imaged with CT followed by static PET scans at 1, 4, and 24 h after tracer administration on an Inveon small animal PET/CT scanner (Siemens Medical Solutions, Malvern, PA, USA). Static images were collected for 20 min and reconstructed with the maximum a posteriori (MAP) reconstruction algorithm using the Inveon Reseach Workstation image display software (Siemens). Regions of interest (ROI) were selected based on co-registered anatomical CT images, and the associated radioactivity was measured using the Inveon software. The maximum standard uptake value (SUV) was calculated as the highest regional radioactivity concentration (nCi/cc) × animal weight (g)/decay-corrected amount of injected dose (nCi).

### Statistical analysis

Quantitative data were processed by Prism 9 (GraphPad Software, La Jolla, CA, USA) and expressed as Mean ± SD. Statistical analysis was performed using one-way analysis of variance and Student’s *t*-test. Differences at the 95% confidence level (*p* < 0.05) were considered statistically significant.

## SUPPLEMENTARY MATERIALS



## Abbreviations

DOTA: (S-2-(4-isothiocyanatobenzyl)-1,4,7,10-tetraazacyclododecane-tetraacetic acid)
PCTA: (3,6,9,15-tetraazabicyclo[9.3.1]pentadeca-1(15),11,13-triene-4-S-(4-isothiocyanatobenzyl)-3,6,9-triacetic acid)
MFU: mean fluorescence unit
