# Supplementary material for: A comparison of 64Cu-labeled bi-terminally PEGylated A20FMDV2 peptides targeting integrin ανβ6
Source: Oncotarget. 2022 Feb 16;13:360–72. doi: 10.18632/oncotarget.28197 (PMC8849274; doi:10.18632/oncotarget.28197)
Supplement: Supplementary file 1 [file oncotarget-13-28197-s001.pdf]

A comparison of ^{64}Cu-labeled bi-terminally PEGylated A20FMDV2 peptides targeting integrin $\alpha_v\beta_6$

SUPPLEMENTARY MATERIALS

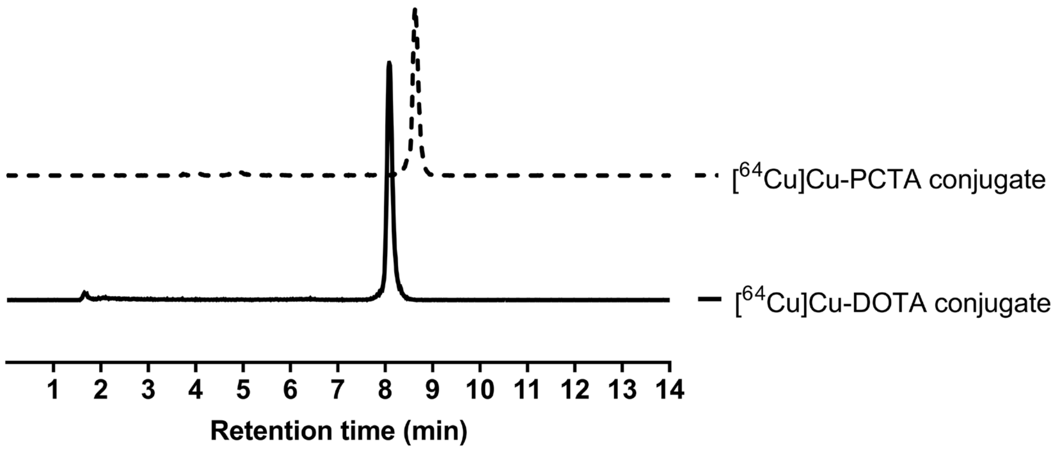

Supplementary Figure 1: HPLC chromatograms showing the ^{64}Cu labeled radiotracers. Radio-HPLC analysis was performed with a mobile phase of water (0.1% TFA) and acetonitrile (0.1% TFA), 20−100% acetonitrile from 0 to 14 mins, and elution was run with a 1 mL/min flow rate.

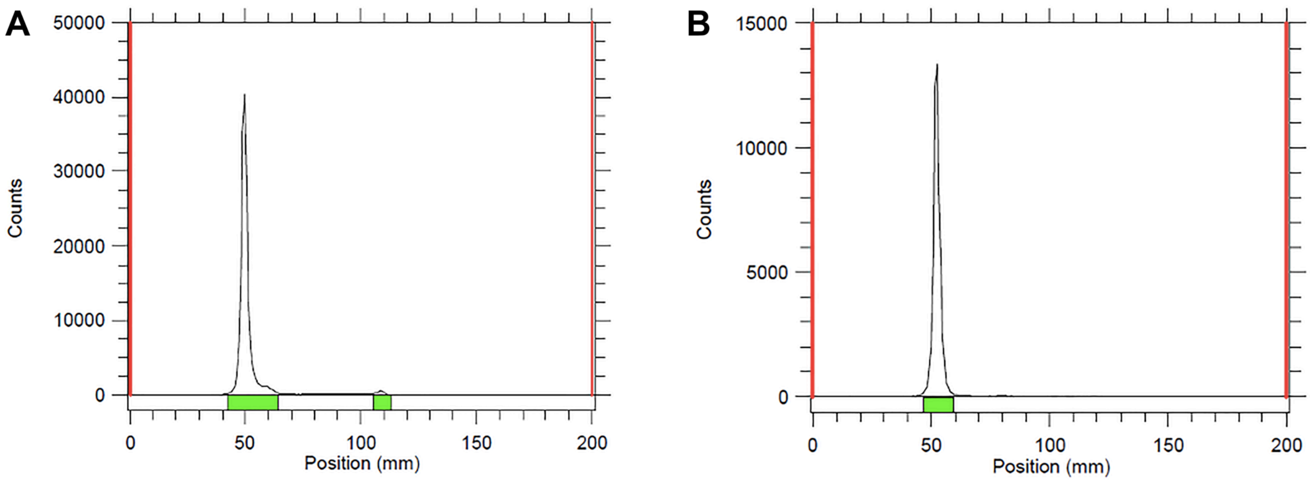

Supplementary Figure 2: Radio-TLC chromatograms of [^{64}Cu]Cu-DOTA-(PEG28)$_2$-A20FMDV2 (**A**) and [^{64}Cu]Cu-PCTA-(PEG28)$_2$-A20FMDV2 (**B**) in 50 mM DTPA.

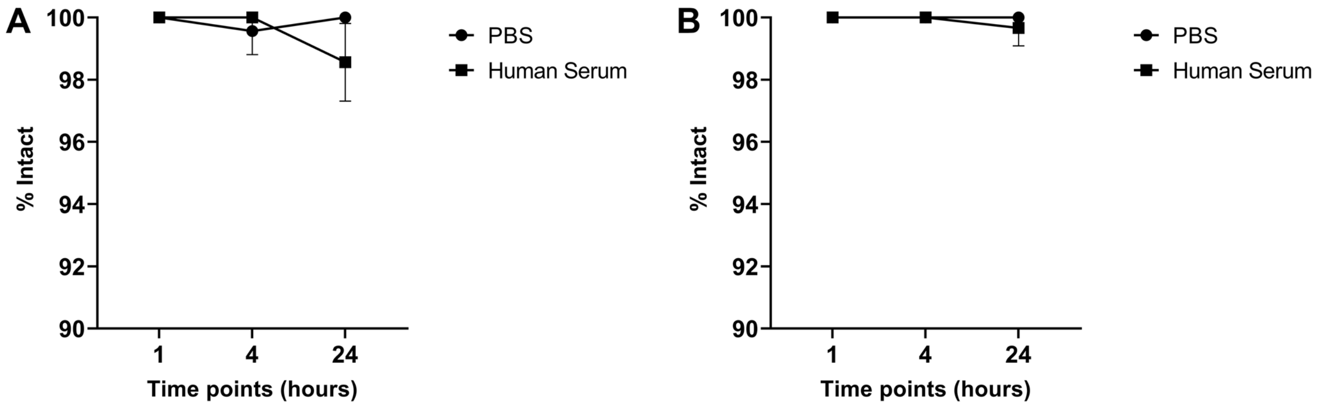

Supplementary Figure 3: Stability profiles in PBS and human serum at 37°C up to 24 h of radiotracers (**A**) [^{64}Cu]Cu-DOTA-(PEG28)$_2$-A20FMDV2 and (**B**) [^{64}Cu]Cu-PCTA-(PEG28)$_2$-A20FMDV2.

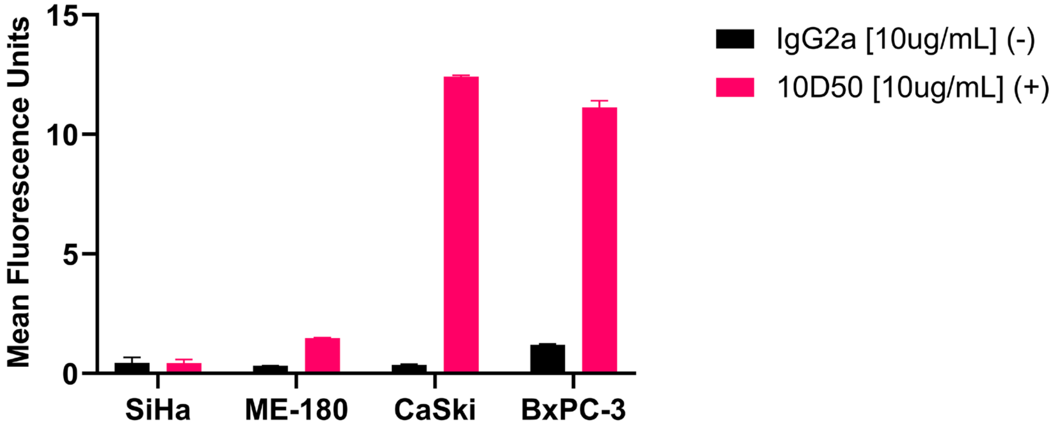

Supplementary Figure 4: Expression of $\alpha_v\beta_6$ on a variety of cells lines as determined by flow cytometry. The bar graph shows mean fluorescence units (MFU) when cells were incubated with either the 10D5 antibody ($\alpha_v\beta_6$-positive) or an IgG2a negative control isotype antibody.

Supplementary Table 1: Tumor-to-organ ratios of ^{64}Cu-labeled bi-terminally PEGylated A20FMDV2 peptides in $\alpha_v\beta_6$-positive CaSki tumor-bearing mice

	1 h after injections				4 h after injection				24 h after injection			
	DOTA		PCTA		DOTA		PCTA		DOTA		PCTA	
	Ratio	SD	Ratio	SD	Ratio	SD	Ratio	SD	Ratio	SD	Ratio	SD
**Blood**	4.87	1.48	2.98	0.29	4.80	0.94	3.09	1.83	2.63	1.07	2.96	0.99
**Lung**	0.71	0.21	0.44	0.15	0.52	0.13	0.61	0.32	0.66	0.18	0.78	0.40
**Liver**	0.90	0.18	0.24	0.06	0.45	0.11	0.34	0.30	0.48	0.17	0.44	0.22
**Spleen**	5.81	0.77	2.56	0.76	3.11	0.47	2.28	1.39	1.72	0.51	1.70	0.49
**Muscle**	1.36	0.30	1.33	0.08	1.71	0.19	2.57	2.15	2.66	1.03	2.59	0.86
**Heart**	1.37	0.34	0.65	0.06	1.48	0.09	1.53	1.25	1.30	0.45	1.21	0.75
**Bone**	3.36	2.59	3.20	1.09	5.51	189	5.22	2.88	5.89	3.17	3.02	1.22
**Kidney**	0.02	0.01	0.01	0.001	0.01	0.002	0.02	0.01	0.04	0.01	0.07	0.05

Supplementary Table 2: Tumor-to-organ ratios of ^{64}Cu-labeled bi-terminally PEGylated A20FMDV2 peptides in $\alpha_v\beta_6$-positive BxPC-3 tumor-bearing mice

	1 h after injections				4 h after injection				24 h after injection			
	DOTA		PCTA		DOTA		PCTA		DOTA		PCTA	
	Ratio	SD	Ratio	SD	Ratio	SD	Ratio	SD	Ratio	SD	Ratio	SD
**Blood**	7.86	2.21	14.25	0.86	5.53	1.23	13.65	2.44	5.49	1.37	9.07	4.49
**Lung**	1.21	0.40	1.51	0.21	1.12	0.14	1.71	0.27	1.31	0.19	1.44	0.45
**Liver**	0.76	0.23	2.02	0.44	0.63	0.11	1.29	0.05	0.64	0.07	1.07	0.28
**Spleen**	7.06	2.08	14.36	3.70	4.21	0.92	11.45	3.39	2.93	0.28	7.60	4.24
**Muscle**	3.37	0.68	3.51	0.78	4.60	0.43	3.75	0.18	5.38	1.12	4.84	0.60
**Heart**	2.06	0.57	2.04	0.32	2.62	0.21	2.72	0.25	2.14	0.34	2.67	0.75
**Bone**	9.29	2.53	10.94	6.20	8.31	1.67	19.22	9.75	7.71	2.66	10.97	5.97
**Kidney**	0.06	0.03	0.04	0.01	0.05	0.01	0.03	0.01	0.09	0.02	0.10	0.07

Supplementary Table 3: Biodistribution profile of ^{64}Cu-labeled bi-terminally PEGylated A20FMDV2 peptides in $\alpha_v\beta_6$-positive CaSki tumor-bearing mice (1 h and 1 h Block)

	DOTA				PCTA			
	1 h		1 h Block		1 h		1 h Block	
	%ID/g	SD	%ID/g	SD	%ID/g	SD	%ID/g	SD
**Blood**	0.22	0.14	0.13	0.02	0.54	0.15	0.76	0.30
**Lung**	1.37	0.36	1.19	0.22	3.76	0.66	1.43	0.26
**Liver**	1.04	0.14	0.94	0.16	6.81	1.67	3.04	1.32
**Spleen**	0.17	0.06	0.13	0.02	0.63	0.05	0.54	0.14
**Muscle**	0.73	0.33	0.51	0.10	1.23	0.04	0.43	0.25
**Heart**	0.71	0.24	0.58	0.04	2.46	0.58	0.55	0.07
**Bone**	0.62	0.76	0.27	0.19	0.54	0.15	0.39	0.08
**Kidney**	42.72	4.03	35.48	4.38	113.58	38.31	43.56	5.63
**Tumor**	0.95	0.29	0.65	0.17	1.63	0.53	0.67	0.26

Supplementary Table 4: Biodistribution profile of ^{64}Cu-labeled bi-terminally PEGylated A20FMDV2 peptides in $\alpha_v\beta_6$-positive BxPC-3 tumor-bearing mice (1 h and 1 h Block)

	DOTA				PCTA			
	1 h		1 h Block		1 h		1 h Block	
	%ID/g	SD	%ID/g	SD	%ID/g	SD	%ID/g	SD
**Blood**	0.28	0.07	0.24	0.07	0.27	0.03	0.27	0.11
**Lung**	1.73	0.29	1.14	0.20	2.58	0.41	1.25	0.27
**Liver**	2.79	0.59	2.59	0.36	1.94	0.20	1.68	0.20
**Spleen**	0.30	0.06	0.34	0.10	0.28	0.04	0.24	0.05
**Muscle**	0.62	0.22	0.18	0.06	1.13	0.24	0.28	0.12
**Heart**	1.01	0.15	0.34	0.06	1.90	0.25	0.27	0.02
**Bone**	0.25	0.12	0.28	0.14	0.38	0.31	0.66	0.92
**Kidney**	38.04	5.47	27.60	2.79	105.39	13.58	80.64	15.50
**Tumor**	2.13	0.83	0.71	0.16	3.86	0.58	0.75	0.09
